# Changes in Intestinal Permeability Ex Vivo and Immune Cell Activation by Three Commonly Used Emulsifiers

**DOI:** 10.3390/molecules25245943

**Published:** 2020-12-15

**Authors:** Elin Oscarsson, Tim Lindberg, Kathrin S. Zeller, Malin Lindstedt, Daniel Agardh, Åsa Håkansson, Karolina Östbring

**Affiliations:** 1 The Diabetes and Celiac Disease Unit, Department of Clinical Sciences, Lund University, 20213 Malmö, Sweden; daniel.agardh@med.lu.se; 2 Department of Immunotechnology, Lund University, 22387 Lund, Sweden; tim@sewe.se (T.L.); kathrin.zeller@immun.lth.se (K.S.Z.); malin.lindstedt@immun.lth.se (M.L.); 3 Department of Food Technology, Engineering and Nutrition, Lund University, 22100 Lund, Sweden; asa.hakansson@food.lth.se (Å.H.); karolina.ostbring@food.lth.se (K.Ö.)

**Keywords:** food emulsifiers, ussing chambers, caco-2, intestinal permeability, polysorbate 80, carboxymethyl cellulose, beta-lactoglobulin, TEER, dendritic cell activation

## Abstract

Food additives such as emulsifiers are used in increasing quantities in the food industry. The aim of this study was to compare three different emulsifiers (polysorbate 80 (P80), carboxymethyl cellulose (CMC), and β-lactoglobulin (β-lac) with regards to their effect on the stimulation of immune cells and intestinal permeability. The immune stimulatory effects were studied in the myeloid cell line MUTZ-3-cells, while the change in intestinal permeability was studied in the Caco-2 cell line and ex vivo in the Ussing chamber system using small intestinal fragments from rats. The tested concentrations of the emulsifiers ranged from 0.02% up to 1%, which are concentrations commonly used in the food industry. The results showed that P80 affected both the myeloid cells and the intestinal permeability more than CMC (*p* < 0.05) and β-lac (*p* < 0.05) at the highest concentration. CMC was found to neither affect the permeability in the intestine nor the MUTZ-3 cells, while β-lac changed the permeability in the total part of the small intestine in rats. These findings indicate that P80 might be more cytotoxic compared to the other two emulsifiers.

## 1. Introduction

The prevalence of inflammatory bowel diseases (IBD), autoimmune diseases, and food allergies have increased worldwide, but especially in industrialized countries [1]. The increase has been linked to an adaptation to the Western lifestyle with, for example, a higher consumption of foods rich in sugar and fat but with low levels of dietary fibers [2]. An increased consumption of emulsions has also been linked to the Western diet, and many of our most popular foods are in emulsion form, such as beverages, mayonnaise, salad dressings, ice cream, and sauces. Therefore, an increase in the industrial use of emulsifier additives has been observed over the decades [2]. Emulsions are a mixture of two immiscible liquids, i.e., oil and water where one is dispersed into the other in the form of small droplets and these droplets need to be stabilized by an emulsifier to enable a homogenous dispersion.

Due to the prolongation of the phase separation, emulsifiers enable an extended shelf life of the food product. However, emulsions are by nature unstable food formulations and the oil droplets will eventually be subjected to coalescence or other destabilization processes, leading to phase separation between the continuous phase (e.g., water) and the dispersed phase (e.g., oil) [3]. Emulsifiers can be divided into two general categories: Synthetic and natural. Polysorbate 80 (P80) is a small, non-ionic surfactant of synthetic origin, stabilizing emulsions mainly by steric repulsion and favorable conditions include acidic pH and the presence of salt [4]. P80 is commonly used in varying concentrations up to 1% in pharmaceuticals and foods such as ice cream and soups [5]. Carboxymethyl cellulose (CMC) is a chemically modified carbohydrate, which is widely used as an emulsifier together with a protein, and the stabilizing effect is suggested to originate from the improved electrostatic and steric repulsion [6]. CMC is classified as “generally regarded as safe” (GRAS) by the Food and Drug Administration (FDA) and can be used in food stuff such as ice cream and cheese. In contrast to P80 and CMC, beta-lactoglobulin (β-lac) is a natural protein found in milk that stabilizes fat droplets in the milk by exposing the hydrophobic α-helices to the oil surface [7].

Emulsifiers have previously been studied in relation to inflammatory bowel diseases such as Crohn’s disease [8,9] where it has been proposed that emulsifiers contribute to Crohn’s disease by inducing bacterial translocation [9], a process which may also increase the risk of developing autoimmune diseases such as celiac disease. Therefore, it has been suggested that the safe consumption levels should be reviewed for children at risk [10].

Studying the relationship between the intake of two commonly used emulsifiers (P80 and CMC) and changes in the gut microbiota and metabolic syndromes in mice has provided support for the hypothesis that the observed increase in IBD, autoimmune diseases, and allergies can be connected to the ingestion of emulsifiers [5]. P80, CMC, and β-lac are emulsifiers representing three different classes of molecules (synthetic, chemically modified, and natural), and due to their different structure and function in emulsions, it can be suspected that they have different mechanisms of action also in the gastrointestinal tract during digestion. Furthermore, P80 has been shown to increase the transport rate of *Escherichia coli* through the mucin layer in the intestinal wall, while CMC and mucin has been shown to interact with each other, forming a network with reduced pore size [11]. Additionally, it has been demonstrated that P80 and CMC altered the composition of the gut microbiota and thereby the thickness of the mucus layer [12].

The permeability of molecules through the intestine is regulated mainly through two different pathways, the paracellular pathway that specifically regulates diffusion of small molecules and the transcellular pathway used mainly for larger molecules, which are transported by transcytosis [13]. There is also evidence suggesting that different parts of the small intestine have different permeabilities, somewhat depending on the properties of the absorbed substance but also on the physiology of the intestine [14]. Several methods have been used to study permeability changes in the intestines, in which the Ussing diffusion chambers are one [15] and cell lines are another.

The human colon adenocarcinoma-derived cell line Caco-2 is frequently used as a model for the intestinal barrier function as they offer transport and permeability characteristics resembling the human intestinal tissue [16,17]. Caco-2 cells can differentiate from a colonic to a small intestinal-like phenotype [18]. However, monocultures based on immortalized cell lines have limitations. Therefore, co-culture models integrating different cell types, e.g., dendritic cells (DCs) and Caco-2 are sought to be developed [19].

DCs are a heterogenous group of immune cells with an important role both in innate and adaptive immune responses. Several sub-populations exist, and they can both lead to tolerogenic and activating responses. In the intestine, DCs are found in the lamina propria and in lymphoid tissues, e.g., the mesenteric lymph nodes and Peyer’s patches [20]. Several DC-like in vitro cell culture models exist, one of them being MUTZ-3 cells. This myeloid cell line is well-characterized and has been shown to adopt gene expression profiles and phenotypes similar to immature and mature DCs upon differentiation and activation [21,22]. MUTZ-3 cells are capable of presenting antigens through CD1d, MHC class I and II, and they can induce specific T-cell proliferation [23].

Additionally, Ussing diffusion chambers have been used to study changes in intestinal permeability induced by various substances [24,25]. Briefly, an intestinal segment is mounted in a diffusion cell and the concentration change over the membrane is determined with marker molecules, such as ions, sugars, larger carbohydrates, and proteins. Furthermore, Ussing chambers have the advantage of the possibility to differentiate between the transcellular and paracellular transport pathways through the intestinal segment depending on the size of the marker molecules [26].

The purpose of this work was to compare emulsifiers from three different classes of molecules (P80, CMC, and β-lac) with regards to their effect on cellular models mimicking the intestinal epithelial barrier and DCs, and their effect ex vivo on intestinal permeability in rats.

## 2. Results

### 2.1. Dendritic Cell Activation

**Figure 1:**
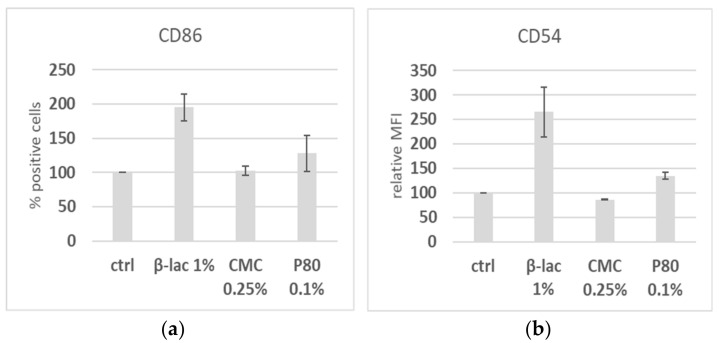
Changes in cell surface expression levels of CD86 (**a**) and the mean fluorescent intensity (MFI) of CD54 (**b**) in MUTZ-3 cells exposed to the indicated substances for 24 h, as determined by flow cytometry (*n* = 2, error bars show standard deviations). Data are normalized to the control cells.

To determine the effect of common food grade emulsifiers on the phenotype of DCs, we assessed the alterations in expression levels of the surface markers CD86, CD54, and HLA-DR on MUTZ-3 cells after 24 h of exposure using flow cytometry. After the titration of concentrations to evaluate the cut-off for non-toxic concentrations, cells were stimulated using several non-cytotoxic concentrations. Figure 1 summarizes the results for the highest used non-cytotoxic concentrations. CMC stimulation did not lead to the upregulation of CD86 or CD54 at any concentration tested (Figure 1 and data not shown), while a trend was observed for the increased CD86 expression levels after P80 stimulation. P80 was cytotoxic down to a concentration of 0.1%, and in samples with a high cell death, CD86 expression was highly upregulated in the remaining viable cell population (data not shown). The β-lac stimulation resulted in almost double the frequency of CD86-positive cells and for 1% compared to the control cells, and also affected the mean fluorescence intensity (MFI) of CD54. No clear effects were observed on the MFI of HLA-DR expression (data not shown). In a subsequent co-culture experiment, where Caco-2 and MUTZ-3 were simultaneously exposed to emulsifiers for 24 h, an investigation of the same markers by flow cytometry followed the same patterns (data not shown).

### 2.2. TEER Measurements

**Figure 2:**
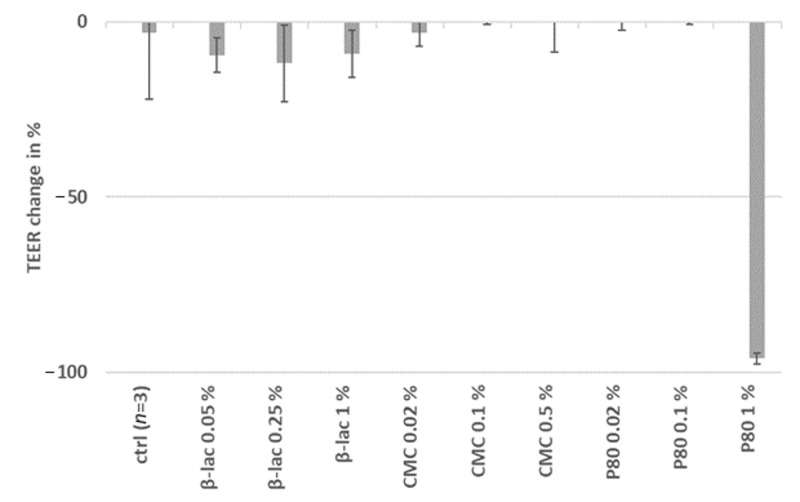
Relative changes in transepithelial electrical resistance (TEER) values after a 24 h exposure to the indicated substances (*n* = 2 except for the control (ctrl), error bars show standard deviations).

Changes in TEER values of Caco-2 cells cultured in transwell inserts after exposure to the three emulsifiers were thereafter measured mimicking the effect of the emulsifiers on the intestinal epithelial cell layer. A drastic decrease in relative TEER values was visible after 24 h of incubation with 1% P80, indicating the disruption of the barrier function of the Caco-2 cell monolayer (Figure 2). In a similar experiment, including MUTZ-3 cells to investigate if the co-culture of the two cell types would influence the Caco-2 monolayer response to the emulsifiers, comparable results were obtained (data not shown).

### 2.3. Passage of Mannitol

Based on the results from the TEER measurements, we hypothesized that exposure to the emulsifiers P80 and β-lac would affect the intestinal barrier function ex vivo. In order to study the change in permeability further, we set up an experiment using Ussing diffusion chambers. Radiolabeled mannitol was used as a marker molecule to study the emulsifiers’ ability to affect the barrier function in the epithelial cell layer with respect to the transport of small molecules in the proximal and distal parts of the small intestine.

When the proximal part of the small intestine was incubated with the different emulsifiers, the passage of mannitol was highest after exposure to 1% P80 after 120 min: 0.34% passage for P80 compared with 0.18% passage for the control, *p* ≤ 0.01 (Table 1). Therefore, the rat intestine was clearly more susceptible to the uptake of the marker molecule in the presence of the food grade emulsifier in industrial approved levels. The passage of mannitol induced by 1% P80 was also significantly higher than the passage induced by CMC (*p* < 0.05) and β-lac (*p* < 0.05) in the same concentration, indicating that different emulsifiers affected the intestinal permeability differently. An increased permeability compared with the control was also observed for 0.75% P80 after 120 min of incubation (0.28% passage; *p* < 0.05), but the other emulsifiers did not affect the permeability in the proximal part of the small intestine (Table 1). When considering the kinetics of the passage by calculating the area under the curve (AUC), it was found that the uptake of mannitol increased to a larger extent for 1% P80 compared to the other emulsifiers investigated. The increased uptake induced by 1% P80 was significantly higher than the uptake for both the control (*p* < 0.01) as well as for CMC and β-lac at the same concentration (*p* < 0.05). There was no significant difference in the uptake induced by 1% CMC and 1% β-lac. When studying the distal part of the small intestine, the only difference in permeability was found for 0.1% P80, which increased the permeability compared with the control (*p* < 0.05) (Table 1).

**Figure 3:**
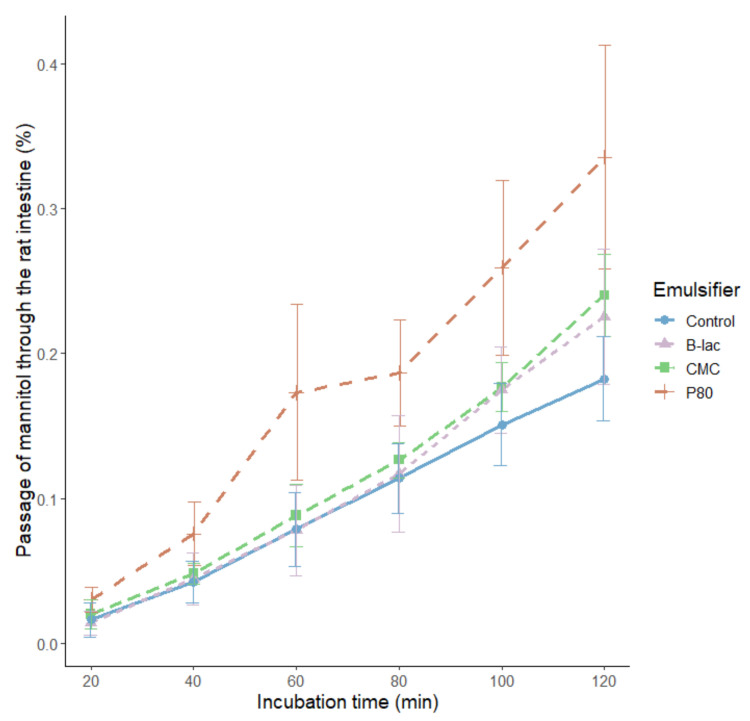
The time-dependent passage of mannitol over the rat intestine in Ussing diffusion chambers (% of the amount added to the mucosal half-cell). The rat intestine was incubated with emulsifiers (1% P80 (*n* = 13), 1% CMC (*n* = 8), and 1% beta-lactoglobulin (*n* = 8)) on the mucosal side for 2 h. Values are represented as mean values, with their corresponding standard error as vertical bars. The increase in the passage induced by 1% P80 was significantly higher than for 1% CMC (*p* = 0.02) and for 1% β-lactoglobulin (*p* = 0.02) calculated based on the AUC.

Since there was no significant difference between the proximal and distal controls (*p* = 0.51), indicating that the natural permeability was the same over the total small intestine, it was possible to combine the results from both the proximal and distal segments. When the permeability of the combined segments was studied, the increased permeability induced by P80 remained, except for the increase seen by 0.1% P80 in the distal part (Table 1). When the intestinal segment was exposed to 1% P80, a higher ratio of mannitol passed through the intestinal wall compared with the other emulsifiers and the control (Figure 3).

**Figure 4:**
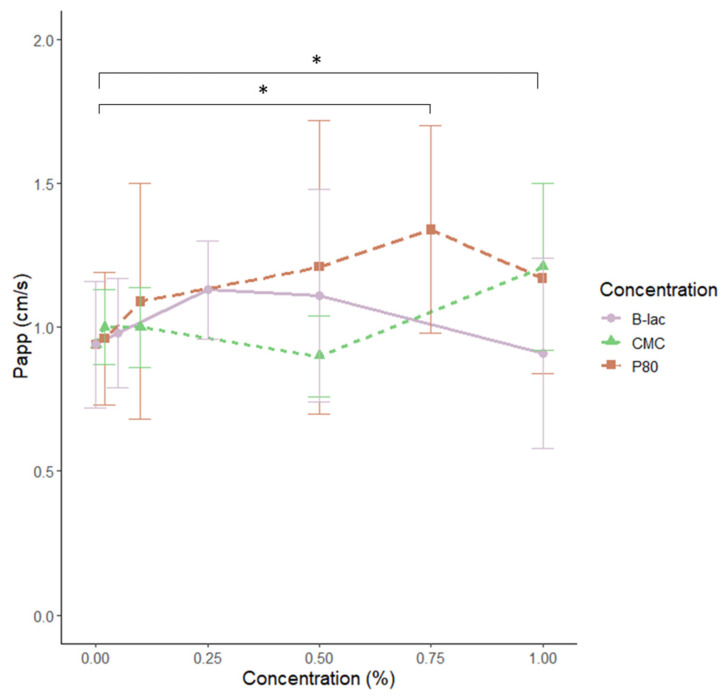
Apparent permeability coefficient (Papp) for the marker molecule mannitol in the small intestine calculated based on the passage of mannitol through the intestine between 60–120 min. The intestinal segments were incubated with the emulsifiers P80 (*n* = 13), CMC (*n* = 8), and β-lactoglobulin (*n* = 8) in different concentrations. The starting value represents the Papp without any added emulsifiers. The values are represented as mean values with standard deviations represented by vertical bars. The emulsifier P80 significantly increased the intestinal permeability at a concentration of 1% (* *p* < 0.05) and 0.75% (* *p* < 0.05).

When studying the apparent permeability coefficient (P_app_) for the different emulsifiers at their respective concentrations, it was shown that 1% P80 and 0.75% P80 both significantly increased the permeability of the intestine (P_app_ = 1.17 cm/s; *p* < 0.05 and P_app_ = 1.34 cm/s; *p* < 0.01, respectively) compared with the control (P_app_ = 0.94 cm/s) (Figure 4). The other emulsifiers did not differ compared with the control.

### 2.4. Macromolecular Permeability

The passage of FITC-dextran through the rat intestine was investigated to determine changes in the permeability for macromolecules after exposure to the different emulsifiers. During the experiment, the passage of FITC-dextran increased in a similar way as mannitol (Table 2). When examining the proximal part of the small intestine, 1% P80 significantly increased the passage of FITC-dextran over time compared with the control (*p* < 0.05). An increase in permeability was also observed for 0.02% P80 (*p* < 0.05). Additionally, a similar increase was seen for 0.02% P80 in the distal part of the small intestine (*p* < 0.05). Furthermore, there was no significant difference between the proximal and distal controls (*p* = 0.26), indicating that the natural permeability was the same over the small intestine and it was possible to combine the results from both parts. However, when studying the total small intestine, there was no significant difference in the passage between 1% P80, 1% CMC, and 1% β-lac, which was seen for mannitol. It seems that larger molecules (>400 Da) had a more pronounced effect on the intestinal permeability in the upper part of the rat intestine. On the contrary, there was an increase in the permeability induced by the two lowest concentrations of P80 compared with the control (0.02% P80; *p* < 0.01 and 0.1% P80; *p* < 0.05). Furthermore, when calculating the apparent permeability coefficient, there was an increased permeability of the intestine induced by P80 0.02% (*p* < 0.05). The concentration of ovalbumin was below the detection limit of the used method, and therefore, could not be analyzed.

## 3. Discussion

Emulsifiers are becoming an increasing part of the diet, especially in industrially produced foods where they are used in the stabilization of emulsions such as sauces, soups, and dressings. In this study, we investigated a model for the immune activation and the change in intestinal permeability induced by three food emulsifiers from different categories. Polysorbate 80 (P80) is a synthetic non-ionic surfactant, which is small and relatively effective compared with carboxymethyl cellulose (CMC), a chemically modified carbohydrate and β-lactoglobulin (β-lac), a natural milk protein with emulsifying properties. In the present study, we have shown that there is a significant difference between P80, CMC, and β-lac with respect to their ability to activate or harm the cellular models and influence the permeability in rat intestinal segments. From the experiments, it was clear that 1% P80 was cytotoxic and that the two highest concentrations (1% and 0.75% P80) increased the permeability of the intestine ex vivo. The ability of P80 to pass through the mucin layer to the epithelial cells has previously been reported by Lock et al. [11] and it might be possible that our findings of cytotoxicity and increased permeability induced by P80 depend on the ability to pass through the mucin layer to the epithelial cells, and by disrupting the cell barrier. In a study by Chassaing et al., P80 at a concentration of 1% was found to influence the gut microbiota and subsequently induce the metabolic syndrome in mice [5]. In our study, the effect of the gut microbiota was not investigated but it was possible to see significant changes in permeability after only 2 h of incubation in the Ussing diffusion chambers. Those results indicate that the emulsifiers themselves could directly impair the integrity of the intestinal barrier and not only indirectly affect the gut through the gut microbiota. Furthermore, P80 at its highest concentration (1%) was shown to be cytotoxic, likely by damaging the cellular membranes. An experiment conducted by Bu et al. showed that P80 at concentrations of 0.5% significantly decreased the viability of Caco-2-cells in a dose dependent way after a 2 h incubation in 1- and 5-day cultures [27], which is in concordance with our results where the most pronounced change was detected at 1% P80. At a concentration of 0.1%, P80 still seemed to increase the number of CD86 positive cells and led to some increase in the MFI of CD54, indicating the activation of the DC model, but this could not formally be shown for the higher P80 concentration due to the strong cytotoxic effect on the cell model. However, the few cells still alive were all positive for CD86 expression (data not shown).

Furthermore, it was demonstrated that P80 was the only emulsifier that induced changes in both the proximal and total part of the rat intestine in this study. The permeability in the intestine normally changes along the length depending on physiological differences in the proximal and distal parts [14]. In our experiments, however, it was possible to study and evaluate the permeability along the total rat intestine, since there was no difference in permeability between the proximal and distal controls. The emulsifier-induced increase in intestinal permeability for the marker molecules mannitol and FITC-dextran was most pronounced in the proximal part of the rat intestine, indicating that the proximal part was more sensitive to changes in the intestinal integrity, thus a larger amount of marker molecules could pass over the intestinal wall. This effect might be explained by the physiological differences found between different parts of the intestine such as the mucus layer. The intestinal epithelial cells are protected from bacteria and other antigens by the mucus layer, in which the thickness varies depending on the position along the intestine. Miyazaki et al. reported that after destruction of the mucus layer, the permeability of lipophilic molecules increased in the proximal part of the intestine, but not in the distal part [14], showing that the permeability of the intestine was affected differently by a disrupted mucus layer. In the present study, the most significant changes in the increased permeability were detected in the proximal part of the small intestine and could therefore possibly be explained by an altering effect on the mucus layer.

The emulsifier CMC has previously been reported to induce a change in the structure of mucin by decreasing the pore size from 109.45 to 59.3 nm [11], but in our study, the intestinal permeability was not affected by CMC at any of the concentrations investigated. One possible reason why our results did not show a decreased permeability, as could be expected based on the findings by Lock et al., could be that the pores in the mucus layer after incubation with CMC were still large enough to allow the passage or uptake of the marker molecules through the epithelial cells. Former studies indicate that CMC affects the conditions in the intestines, but this might be an indirect effect of a changed bacterial composition and not a direct alteration in the intestinal permeability. As mentioned earlier, Chassaing et al. reported that both P80 and CMC changed the bacterial composition and induced the metabolic syndrome in mice [5], and both emulsifiers have also been found to induce a gut dysbiosis that favors tumor development in a model system for colorectal cancer [28].

No change was seen in the TEER values after incubation of Caco-2 with CMC, nor did CMC activate the DC-model. β-lac, on the other hand, seemed to activate cells as indicated by the increase of CD86 positive MUTZ-3 cells and increased MFI for CD54, but no changes were observed on the TEER values using the Caco-2 model. Although β-lac preparations were sterile-filtered before use in the in vitro experiments, a contribution of endotoxin to the activating effect of β-lac cannot be excluded. The immunostimulatory potential of β-lac, regarded as one of the major allergens in cow’s milk has been described earlier. However, it has also been proposed that this immunostimulatory capacity actually is caused by an endotoxin contamination of certain commercially available β-lac preparations [29].

Interestingly, β-lac at concentrations of 0.25% and 0.5% had a significant effect on the permeability of mannitol in the small intestine, but only when analyzing both the proximal and the distal part together. This might indicate that β-lac affects both the proximal and distal part of the rat intestine to a limited extent, but that the effect is only detectable when studying both parts in a combined model. This change in permeability induced by β-lac is supported by Marcon-Genty et al., who reported that β-lac affected the intestinal permeability investigated in the Ussing chamber system [30]. In the present study, β-lac only affected the permeability of the smallest marker molecule (mannitol) and that might indicate that the effect on the pore size induced by β-lac was relatively small. Therefore, the permeability of the larger marker molecule FITC-dextran remained unaffected in the presence of β-lac on the mucosal side of the intestine.

Furthermore, there has been evidence suggesting that the response to an emulsifier is very individual and that the inter-individual variation needs to be further studied [31]. In our experiments, we observed some indications that rat intestinal segments from different individuals responded differently to the same emulsifier at a given concentration, which is reflected in the large variation in the data set (Figure 3 and Figure 4). However, despite the individual variations, we found that there was a clear difference between P80 and the other two emulsifiers regarding their ability to stimulate the myeloid cells and induce changes in the intestinal permeability in the rat (*p* < 0.05). This could potentially be due to the fact that the emulsifiers tested belong to different classes, stabilize emulsions in different ways, and subsequently affect the intestines differently based on their chemical properties.

## 4. Materials and Methods

### 4.1. Cell Culture

The human myeloid leukemia-derived cell line MUTZ-3 (DSMZ, Braunschweig, Germany) was cultivated in MEM/alpha-modification with l-glutamine (Nordic Biolabs/Gibco) supplemented with 20% (*v*/*v*) fetal calf serum (Life Technologies, Carlsbad, CA, USA) and 40 ng/mL recombinant human GM-CSF (Peprotech, Rocky Hill, NJ, USA), as described [22]. The human colorectal adenocarcinoma-derived cell line Caco-2 (ATCC, Manassas, VA, USA) was initially cultured in MEM/EBSS (Nordic Biolabs/Gibco) supplemented with 1 × non-essential amino acids, l-glutamine, and penicillin/streptomycin (all Nordic Biolabs) and 20% fetal calf serum (Life Technologies), but culture conditions were switched to RPMI with 10% fetal calf serum (Life Technologies) and l-glutamine (Nordic Biolabs) as no differences in cell growth and morphology were observed.

### 4.2. Cell Activation

To evaluate the potential cytotoxicity of the emulsifiers polysorbate 80 (P80), approximate molecular weight: 79,000, a mixture of sorbitol oleic esters and sorbitanes with polyethylene glycol chains (Sigma-Aldrich, St Louis, MO, USA), carboxymethyl cellulose (CMC), high viscosity sodium salt, degree of substitution 0.7–0.8 per anahydroglucose unit (A. Johansson and Co. H.A.B, Malmö, Sweden) and β-lactoglobulin (β-lac) from bovine milk, approximately 18,400 Da (Sigma-Aldrich, St Louis, USA), MUTZ-3 were exposed to the emulsifiers at five different concentrations (1%, 0.5%, 0.1%, 0.02%, 0.004% for P80 (*w*/*v*), and β-lac (*w*/*v*); 0.5%, 0.25%, 0.05%, 0.01%, and 0.002% (*w*/*v*) for CMC due to the solubility issues in cell media). No significant impact on cell viability as measured by Propidium iodide (BD Biosciences, Franklin Lakes, NJ, USA) was observed after exposure to CMC and β-lac. However, P80 led to the cell death at concentrations ≥0.2%. A more detailed dilutions series confirmed >95% viability for P80 at and below 0.1%.

Subsequently, the DC activation marker expression was investigated by flow cytometry at non-cytotoxic concentrations using two biological replicates, i.e., distinct cell batches. All staining procedures and washing steps were performed using the BSA Cohn fraction V 0.5% (*w*/*v*) in a phosphate buffer solution (PBS). Cells were incubated with the following specific mouse mAbs for 15 min at 4 °C: FITC-conjugated CD86, PE-conjugated CD54, FITC-conjugated HLA-DR (all BD Biosciences, Franklin Lakes, USA), and analyzed using a FACSCanto II instrument (BD Biosciences, Franklin Lakes, USA). FITC- and PE-conjugated mouse IgG1 (BD Biosciences, Franklin Lakes, USA) served as isotype controls. Ten thousand events were acquired, and gating was based on light scatter properties to exclude non-viable cells and debris. Further data analysis was performed using FCS Express V4 (De Novo Software, Los Angeles, CA, USA). Close to 100% of the cells were positive for HLA-DR and CD54, and thus the median fluorescence intensity (MFI) of the control cells was compared with the treated cells for these markers.

### 4.3. Transepithelial Electrical Resistance

The effect of P80, CMC, and β-lac on the barrier function was measured in a cell culture of Caco-2 cells seeded on transwell inserts (Falcon®, 3 µm pore size, Corning, Corning, NY, USA) placed into 12-well plates and grown until confluency. The integrity of the cell layers was studied using transepithelial electrical resistance (TEER), using a Millicell ERS-2 Epithelial Volt-Ohm Meter (Millipore, now MerckMillipore, Burlington, MA, USA). The electrical resistance of a cellular monolayer is widely used as a non-invasive and quantitative measure of the barrier integrity. The cells were grown until the TEER values reached a plateau, indicating that the cells had formed a tight monolayer and were then incubated with the emulsifiers in three different concentrations (P80: 1%, 0.1%, and 0.02% (*v*/*v*), CMC: 0.5%, 0.1%, and 0.02% (*w*/*v*), beta-lactoglobulin: 1%, 0.25%, and 0.05% (*w*/*v*)) in duplicates for 24 h before measuring TEER again. The TEER values are temperature-sensitive, therefore, the media was changed to room-temperature equilibrated media prior to all measurements.

The TEER values, in units of ohm (Ω), were calculated according to Ohm’s Law Method as described by [32]. In short, for each separate measurement the cell-specific resistance (R_tissue_) (1) was calculated by obtaining the blank resistance (R_blank_), which is the resistance across the semipermeable membrane without the cells, and the resistance across the semipermeable membrane when the cells are present (R_total_). Further, TEER is reported as Ω.cm^2^ (TEER_reported_) (2) by multiplication of the R_tissue_ with the effective area of the semipermeable membrane (M_area_), which in this case is 0.3 cm^2^.

R_tissue_ (Ω) = R_total_ − R_blank_ (1)

TEER_reported_ (Ω.cm^2^) = R_tissue_ × M_area_ (2)

The visualization of the change in TEER of different emulsifiers (Figure 3) are presented as the difference (3) of the average of two replicates of TEE_Rreported_ before incubation and TEER_reported_ after 24 h of incubation, where 0% indicates no change in TEER between the two measurements.

(3) $$TEER\%\;change\frac{{\mathrm{Average}\;\mathrm{TEER}}_{\mathrm{reported}}\;\left(\mathrm{after}\;24\;h\;\mathrm{incubation}\right)}{{\mathrm{Average}\;\mathrm{TEER}}_{\mathrm{reported}}\;\left(\mathrm{before}\;\mathrm{incubation}\right)}-1$$

### 4.4. Preparation of Marker Solution for Ex Vivo Studies in Rats

The modified Krebs buffer (0.1 M NaCl, 3 mM CaCl_2,_ 5.5 mM KCl, 14 mM KH_2_PO_4_, 29 mM NaHCO_3_, 5.7 mM sodium-pyruvate, 7 mM sodium-fumarate, 5.7 mM sodium-glutamate, pH 7.4) was prepared and oxygenated with carbogen (O_2_:CO_2_, 95:5, *v*/*v*) for 5 min. d-glucose (13.4 mM) was added to the buffer on the serosal side and mannitol (13.4 mM) was added to the buffer on the mucosal side. The marker solution was prepared by the addition of ovalbumin (25 g/L, 45,000 Da) (Sigma-Aldrich, St Louis, USA), FITC-dextran (1 g/L, 4000 Da) (TdB Consultancy, Uppsala, Sweden), and C^14^ labeled mannitol (2.85 KBq/mL, 190 Da) (Perkin Elmer, Waltham, USA) to a modified Krebs buffer, which were added to the marker solution in commercially commonly used concentrations: P80: 0.02–1% (*v*/*v*), CMC: 0.02–1% (*w*/*v*), and β-lac: 0.05–1% (*w*/*v*).

### 4.5. Animals

The study was performed with a total of 14 female rats (260–300 g) of the Sprague-Dawley stock (Janvier labs, France). The rats were housed two or three per cage on a chopped wood bedding in polycarbonate cages with free access to tap water and pelleted breeding chow. The study was approved by the Lund University Ethical Committee for animal Experiments and conducted according to European Community regulations concerning the protection of experimental animals (Dnr 5.8.18-08514/2017).

The rat was anaesthetized with isoflurane (Shering-Plogh A/S, Bellerup, Denmark). The proximal (20 cm from the ligament of Treitz) and distal (20 cm to the ileocecal ligament) parts of the small intestine were collected, rinsed with a buffer, and immediately immersed in room tempered modified Krebs buffer. In addition, the segments were kept for up to 30 min before the onset of the experiments. The intestines were considered viable for a minimum of 2.5 h after the collection. The animals were sacrificed after the tissue collection.

### 4.6. Mounting

The intestines were cut in 3 cm sections and opened on the opposite side to the mesentery to preserve the blood vessels. The segments were pinched to nails of a pre-heated (37 °C) Ussing half-cell (Precision Instrument Design, Los Altos, CA, USA) modified in accordance with Grass and Sweetana [33]. The half-cell was connected to another half-cell, giving the arrangement two sides: One facing the serosal side and one facing the mucosal side. The exposed intestinal area was 1.78 cm^2^ between the two half-cells. Five milliliters of the modified Krebs buffer was added to each half-cell. The Ussing chambers were then connected to the carbogen supply (O_2_:CO_2_, 95:5, *v*/*v*) and were kept at 37 °C. Gas lift ensured the circulation of the liquid in the Ussing half cells.

### 4.7. Start of the Experiment

The buffer was exchanged to a fresh buffer in the serosal half-cell and prepared marker solution (modified Krebs buffer with marker molecules and emulsifier) or the control solution (modified Krebs buffer with marker molecules) in the mucosal half-cell. Serosal samples of 1 mL were collected at 20, 40, 60, 80, 100, and 120 min, with the replacement of the sampled volume by the fresh buffer at each time point.

### 4.8. Analysis of Marker Molecules

The amount of radio-labeled mannitol was quantified by mixing 0.5 mL of the withdrawn sample with 5 mL scintillation cocktail (Ultima Gold, Perkin Elmer, Waltham, MA, USA) and measured using liquid scintillation. FITC-dextran was quantified by fluorescence spectrophotometry (SpectraMax i3 x, Molecular Devises, San Jose, CA, USA) at an excitation wavelength of 490 nm and an emission wavelength of 520 nm. The marker solution diluted in the modified Krebs buffer was used as a standard. The amount of ovalbumin was determined using an Agilent Bioanalyzer 2100 with the Agilent Protein 230 Kit (Agilent Technologies, Santa Clara, CA, USA) according to the manufacturer’s instructions. Briefly, a 40 µL sample was mixed with 2 µL dye and heated to 95 °C for 5 min. An amount of 6 µL of the mixture was loaded to the protein 230 chip after staining of the gel. The amount was quantitatively determined using a reference solution with the known concentration (800 ng/µL).

### 4.9. Calculations for Permeability Measurements

The uptake of methyl-mannitol was determined as the percentage passing through the intestinal segment at specific time points, by means of the following formula:

(4) $$Uptake\;\left(\%\right)=\frac{\mathrm{ci}}{c0}\;\times \;100$$

where c_i_ represents the concentration of the marker molecule present in the serosal half-cell and c_0_ represents the initial concentration of the same marker molecule in the mucosal half-cell [34].

The apparent permeability coefficient (P_app_) was calculated by using the following formula:

(5) $$P_{\mathrm{app}}\;=\frac{\mathrm{dc}}{\mathrm{dt}}\times \;V\;\times \frac{1}{c0\;\times A}$$

where dc/dt (M/s) represents the change in concentration (M) on the serosal side in the time period from 60 to 120 min. V represents the volume (cm^3^) of the half-cell, c_0_ is the starting concentration (M) on the mucosal side, and A is the area (cm^2^) of the exposed intestine [34].

### 4.10. Statistics

The Student’s t-test was used for the statistical analyses for the passage through the intestine, and the apparent permeability coefficient. The comparison was considered significantly different if *p* < 0.05. The passage, or uptake, of marker molecules over time was determined. The area under the curve (AUC) was calculated for the passage, or uptake, of marker molecules by a numeric integration using the trapezoidal rule method (MATLAB R2017a). The statistics were calculated using Excel 2010 and R version 3.5.1.

## 5. Conclusions

In conclusion, emulsifiers in industrially used concentrations were tested for their ability to stimulate immune cells and affect the permeability through the rat intestine. P80 affected both the permeability of Caco-2 monolayers and the rat intestinal permeability, and there is a tendency to activate the used DC model MUTZ-3 cells, while β-lac activated MUTZ-3 and increased the intestinal permeability of small molecules in the rat. CMC did not affect neither the DC response nor the intestinal permeability. There was a difference between the three emulsifiers where P80 caused most damage, and CMC and β-lac seemed to affect the intestinal permeability to a lower extent ex vivo. In light of these results and the increasing usage of food emulsifiers in the Western diet, there is a need to further investigate those additives and their effects on the intestinal health both in vitro and in vivo. Additionally, the effects on the intestinal health by emulsifiers incorporated in food products needs to be studied.

## Figures and Tables

**Table 1:** The passage of the marker molecule mannitol in both the proximal part, the distal part, and the total (proximal and distal part together) small intestine at 120 min after the start of the experiment for the different emulsifiers at varying concentrations. The values are presented as the mean ± standard deviation together with the number of replicates (*n*). The *p*-values are calculated based on differences in kinetics detected by the AUC analysis compared with the control.

Emulsifier	Concentration (%)	Passage in the Proximal Part		Passage in the Distal Part (%)		Passage in the Total Intestine (%)	
		(%)	(*n*)	(%)	(*n*)	(%)	(*n*)
Control	0	0.21 ± 0.04	20	0.18 ± 0.03	21	0.19 ± 0.04	41
β-lac	0.05	0.19 ± 0.02	2	0.21 ± 0.03	3	0.20 ± 0.04	5
	0.25	0.28 ± 0.02	3	0.22 ± 0.02	3	0.25 ± 0.04 *	6
	0.5	0.28 ± 0.04	3	0.21 ± 0.04	3	0.24 ± 0.05 *	6
	1	0.23 ± 0.05	4	0.16 ± 0.03	4	0.19 ± 0.05	8
CMC	0.02	0.22 ± 0.02	3	0.19 ± 0.03	3	0.21 ± 0.03	6
	0.1	0.19 ± 0.02	3	0.21 ± 0.03	3	0.20 ± 0.03	6
	0.5	0.19 ± 0.04	3	0.17 ± 0.01	3	0.18 ± 0.03	6
	1	0.24 ± 0.03	4	0.27 ± 0.02	4	0.25 ± 0.03	8
P80	0.02	0.25 ± 0.02	3	0.20 ± 0.03	3	0.22 ± 0.04	6
	0.1	0.27 ± 0.05	5	0.22 ± 0.03 *	6	0.24 ± 0.05	11
	0.5	0.24 ± 0.06	3	0.24 ± 0.11	3	0.24 ± 0.09 *	6
	0.75	0.28 ± 0.04 *	4	0.26 ± 0.03	3	0.27 ± 0.04 **	7
	1	0.34 ± 0.08 **	4	0.23 ± 0.04	9	0.26 ± 0.07 **	14

** *p* < 0.01 and * *p* < 0.05 calculated based on the area under the curve (AUC) values.

**Table 2:** The passage of the marker molecule FITC-dextran in both the proximal part, the distal part, and the total (proximal and distal part together) small intestine at 120 min after the start of the experiment for the different emulsifiers at different concentrations. The values are presented as the mean ± standard deviation together with the number of replicates (*n*). The *p*-values are calculated based on differences in kinetics detected by the AUC analysis compared with the control.

Emulsifier	Concentration (%)	Passage in the Proximal Part		Passage in the Distal Part		Passage in the Total Intestine	
		(%)	(*n*)	(%)	(*n*)	(%)	(*n*)
Control	0	0.29 ± 0.08	20	0.23 ± 0.06	20	0.26 ± 0.07	40
β-lac	0.05	0.30 ± 0.04	2	0.36 ± 0.08	3	0.33 ± 0.08	5
	0.25	0.52 ± 0.11	3	0.28 ± 0.03	2	0.46 ± 0.24	5
	0.5	0.36 ± 0.06	3	0.22 ± 0.02	3	0.29 ± 0.08	6
	1	0.31 ± 0.10	4	0.21 ± 0.12	4	0.26 ± 0.12	8
CMC	0.02	0.33 ± 0.06	3	0.30 ± 0.08	3	0.32 ± 0.07	6
	0.1	0.29 ± 0.03	3	0.32 ± 0.07	3	0.30 ± 0.06	6
	0.5	0.31 ± 0.09	3	0.28 ± 0.01	3	0.30 ± 0.07	6
	1	0.33 ± 0.06	4	0.37 ± 0.17	4	0.35 ± 0.06	8
P80	0.02	0.49 ± 0.06 *	3	0.35 ± 0.05 *	3	0.42 ± 0.09 **	6
	0.1	0.38 ± 0.10	4	0.39 ± 0.14	5	0.38 ± 0.12 *	9
	0.5	0.28 ± 0.10	2	0.37 ± 0.14	3	0.33 ± 0.13	5
	0.75	0.30 ± 0.05	4	0.27 ± 0.02	2	0.29 ± 0.04	6
	1	0.39 ± 0.13 *	5	0.27 ± 0.06	9	0.31 ± 0.11	14

** *p* < 0.01 and * *p* < 0.05 calculated based on the AUC values.
